# Suit for Malpractice

**Published:** 1858-01

**Authors:** 


SUIT FOR MALPRACTICE—CAUTION TO SURGEONS.—We do not know when we have had our professional indignation more thorougly aroused toward a so-called court of justice, and our sympathies more warmly enlisted in favor of the unfortunate brother who was the immediate sufferer, than in the perusal of a case that lately transpired in one of the interior towns of this State. It appears from the report published in the newspapers, the main points of which, we are told by a gentleman who was present at the trial, are substantially true, that a man, by an accident, unnecessary here to detail, suffered fracture of the thigh-bone. His family-physician was called in, who, not wishing to take the entire responsibility of the case, sent for another practitioner, Dr. W. McM., to see the case with him in consultation. The latter resided six miles from the patient, and partly on account of sickness in his own person, the *expectant* condition of his wife, and partly on account of the inconvenience of attending an important case so far away, refused to obey the summons. Upon the report of this to the injured man and his physician, they directed the messenger to return to Dr. McM. and say to him that he must come, if only for once. Such was the urgency of the appeal that Dr. McM. consented to go, but with the expressed intention of paying but a single visit. Arriving at the house he positively refused to take charge of the patient, but agreed to dress the limb, which having done with the assistance of the family-physician, he left the case in the hands of the latter. The splints were taken off on the tenth week, and it was proved that up to that time the treatment had been proper in every particular. However, deformity subsequently took place, and the family-physician having left the village, Dr. McM. was prosecuted by the patient, and mulcted in the sum of Five Hundred Dollars and costs. The point which the prosecuting party urged, and upon which the verdict was grounded, was that, a physician or surgeon having once visited a patient, could not discharge himself without the consent of the patient, no matter what the circumstances under which the visit was made, or what the physician's previous intentions or motives; and that, therefore, Dr. McM., through culpable neglect, was responsible for the deformity of the patient's limb.

